# Serum Magnesium Levels in Patients with Obstructive Sleep Apnoea: A Systematic Review and Meta-Analysis

**DOI:** 10.3390/biomedicines10092273

**Published:** 2022-09-14

**Authors:** Zahraa Al Wadee, Soo Liang Ooi, Sok Cheon Pak

**Affiliations:** 1 School of Dentistry and Medical Sciences, Charles Sturt University, Bathurst, NSW 2795, Australia; 2 Smiles Unlimited Dental Clinic, Fairfield, Sydney, NSW 2165, Australia

**Keywords:** intermittent airway obstruction, sleep disorder, micronutrient deficiency, metabolic risk factor, cardiovascular disease

## Abstract

*Aims:* Obstructive sleep apnoea (OSA) affects patients’ quality of life and health. Magnesium (Mg) is an essential mineral and a potent antioxidant. Mg deficiency can worsen oxidative stress caused by sleep deprivation or disorders. The impact of OSA on serum Mg levels and its health consequences remain unclear. *Data Synthesis:* This study systematically reviewed clinical studies investigating the serum Mg levels of OSA patients and the potential relationships with other biomarkers. Six articles were included for qualitative synthesis and quantitative analysis. Two out of four studies that compared OSA patients to healthy controls found them to have significantly lower serum Mg levels. Our meta-analysis with three studies shows that patients with OSA had significantly lower serum Mg with an effect size of −1.22 (95% CI: −2.24, −0.21). However, the mean serum Mg level of OSA patients (*n* = 251) pooled from five studies (1.90 mg/dL, 95% CI: 1.77, 2.04) does not differ significantly from the normal range between 1.82 to 2.30 mg/dL. OSA severity appears to affect serum Mg negatively. Serum Mg levels generally improve after treatment, coinciding with the improvement of OSA severity. Low serum Mg levels correlate with the worsening of cardiovascular risk biomarkers of C-reactive protein, ischaemia-modified albumin, and carotid intima-media thickness. The serum Mg levels also potentially correlate with biomarkers for lipid profile, glucose metabolism, calcium, and heavy metals. *Conclusions:* Sleep deprivation appears to deplete Mg levels of OSA patients, making them at risk of Mg deficiency, which potentially increases systemic inflammation and the risk of cardiovascular and metabolic diseases.

## 1. Introduction

Human beings spend approximately one-third of their lives sleeping. Adequate and restful sleep is essential for maintaining optimal health. Sleep deprivation resulting from functional disorders can lead to poor quality of life and morbidity. Obstructive sleep apnoea (OSA) is characterised by frequent episodes of partial or complete upper airway collapse during sleep. The disruption of respiratory airflow can occur when there is a physical blockage to the airways caused by the tongue and surrounding soft tissue structures falling back to the throat due to gravity and muscle relaxation. These episodes result in complete or partial reduction of airflow and recurrent arousals from sleep [1,2].

The pathogenesis of OSA is multifactorial, with a combination of both anatomical and non-anatomical causes. OSA patients often have pharyngeal anatomical abnormalities such as narrow pharyngeal airway, increased airway length, and specific pharyngeal lumen shapes. Non-anatomical factors, such as impaired pharyngeal dilator muscle function, low respiratory arousal threshold, and unstable control of breathing, also contribute to upper airway collapse and frequent arousal during sleep [1]. Individuals with OSA may experience symptoms such as snoring, excessive daytime sleepiness, morning headache, fatigue, non-refreshing sleep, nocturia, irritability, and memory loss [1,3]. Without proper management, OSA can severely impact their health-related quality of life.

Magnesium (Mg) is an essential mineral for biochemical activities in the human body and is involved in many enzyme systems as a cofactor [4]. In mammals, cells regulate Mg content through the controlled transport and buffering mechanisms maintained by various hormones and cellular messengers. A dynamic relationship exists between the changes in the total content of Mg transported across cells and within cellular compartments through the effect of hormones and agents [5]. Hence, it is essential to maintain an adequate cellular Mg level for optimal enzyme function in energy metabolism and neurotransmitter synthesis [6]. Mechanisms associated with impaired Mg regulation can be related to common chronic diseases, which can also be affected by dietary intake. Current dietary guidelines recommend a daily intake of 310–420 mg of Mg to maintain health and lower the risk of inflammatory diseases [7]. However, in Australia, 37% of males and 34% of females did not meet the daily requirements for Mg and may be at risk of Mg deficiency [8]. Signs of Mg deficiency are non-specific, which may include low potassium and calcium levels, elevated blood pressure, neuromuscular irritability, muscle cramps and spasms, as well as mental disturbances such as depression, confusion, agitation, and hallucinations [9].

Serum Mg level is widely used in clinical practice to assess the total body Mg status. The commonly used reference range for serum Mg level is between 1.82 to 2.30 mg/dL (0.75–0.95 mmol/L) based on the 1974 National Health and Nutrition Examination Survey 1 (NHANES 1) [10]. This range was derived from the central 95th percentile of serum Mg levels in 15,820 healthy individuals aged 18–74 with an estimated mean of 2.07 mg/dL (0.85 mmol/L). Such a reference range does not truly reflect the relationship between serum Mg levels and clinical outcomes. Hence, having a level of serum Mg within the reference range does not rule out the possibility of subclinical or chronic latent deficiencies [11].

Nonetheless, serum Mg remains a practical and valuable biomarker for assessing Mg status when used in combination with 24-h urinary Mg excretion and dietary Mg intake [10]. A recent evidence-based recommendation proposed that the clinical cut-off of serum Mg should be at 2.07 mg/dL (0.85 mmol/L). The suggested healthy range of serum Mg level is between 2.07 to 2.30 mg/dL (0.85–0.95 mmol/L). Anyone with serum Mg < 2.07 mg/dL is at risk of moderate to severe Mg deficiency and an increased risk of chronic conditions such as diabetes mellitus [11].

Deficiency in Mg intake from dietary and subsequent Mg inadequacy have been associated with many chronic conditions, including cardiovascular diseases [12], metabolic syndrome [13], diabetes mellitus [14], and obesity [15]. Similarly, being overweight or obese and metabolic syndrome also increase the risk of having moderate to severe OSA [16], which is further linked to hypertension, diabetes, coronary artery disease, heart failure, and cardiac arrhythmias [17]. Hence, Mg deficiency and OSA share common metabolic comorbidities as Mg is a cofactor in many fundamental functions, including energy production, glycaemic control, myocardial contraction, and blood pressure [18]. Notwithstanding, the link between Mg level and OSA remains poorly understood.

This study aims to systematically search and review the published literature to assess the relationship between serum Mg and OSA to answer the following research questions. In adult patients diagnosed with OSA: (1) Do they have lower than normal serum Mg levels? (2) Are they at risk of Mg deficiency based on their serum Mg levels? (3) Are there any correlations between serum Mg level with OSA severity? (4) Do their serum Mg levels correlate with other metabolic, nutritional, inflammatory, or cardiac markers?

## 2. Materials and Methods

We conducted systematic searches on PubMed, EBSCOhost (Health), ProQuest, and Web of Science between May and June 2020 and again from November to December 2020. The keywords used in the searches are: (magnesium) AND ((obstructive sleep apnoea) OR (obstructive sleep apnea) OR (sleep apnoea) OR (sleep apnea) OR (intermittent airway obstruction) OR (sleep disordered breathing)). No restriction was placed on the publication date. We also manually searched the references of the full-text articles downloaded during the review process. The searches were conducted by two authors (Z.A.W. and S.L.O.) independently. Search results were downloaded and imported in EndNote X8.2 to remove duplicates, screen and manage articles. A study protocol was prepared internally before the search and data extraction. The review protocol was not registered on PROSPERO or any other public registry. The PRISMA 2020 checklist of this review is available for download in the Supplementary Materials section below.

### 2.1. Selection Criteria

Criteria of inclusion: (1) human study, (2) published in English, (3) participants were adults (≥18 years old), (4) diagnosed with OSA with apnoea–hypopnoea index (AHI) > 5/h, and (5) the study measured serum Mg levels before any intervention. Two authors (Z.A.W. and S.L.O.) conducted study selection, and the third author reviewed the results (S.C.P.).

### 2.2. Data Extraction and Meta-Analysis

We extracted the following information from the study: first author, year of publication, country, participants’ characteristics, sample size and gender distribution, study design, mean serum Mg in mg/dL with the standard deviation (SD) of the OSA patients and controls (if any), and relevant findings of the study.

For studies that compared the serum Mg of OSA patients to healthy controls, the sample sizes of OSA and control groups and the standardised mean difference (Hedge’s g) with SDs of both groups were combined for meta-analysis. We also performed a meta-analysis of single means by pooling the sample sizes, mean serum Mg levels and SDs of the cross-sectional (or baseline) measurements across all studies. All analyses were done based on published data only. For studies that did not report the SDs, the *p*-value or 95% confidence interval (CI) was used for estimation using the RevMan Calculator [19]. The data from each study were weighted such that studies with less variance or a larger sample size contributed more heavily to the overall estimate of means under the inverse variance method. The heterogeneity between studies was examined using I${}^{2}$ statistics, with 25%, 50%, and 75% values reflecting low, moderate, and high heterogeneity. The random effects model was adopted for the meta-analysis to compensate for the heterogeneity across studies. We investigated the source of heterogeneity through the elimination of studies. The ‘meta’ and ‘metafor’ packages in R (version 4.03) were used to perform the meta-analysis and display results. The Student’s *t*-test was used for testing the significant difference between the pooled mean and the known population mean with the significance level set at 0.05. Z.A.W. and S.L.O. performed data extraction followed by meta-analysis, and S.C.P. reviewed the results.

### 2.3. Assessment of Methodological Quality

The study adopted the Quality Assessment Tool for Case-Control, Observational Cohort and Cross-Sectional Studies published by the National Institute of Health for quality assessment of the included studies [20]. All authors rated the studies independently. Differences in rating were resolved through consensus after discussion.

## 3. Results

### 3.1. Search Results

**Figure 1:**
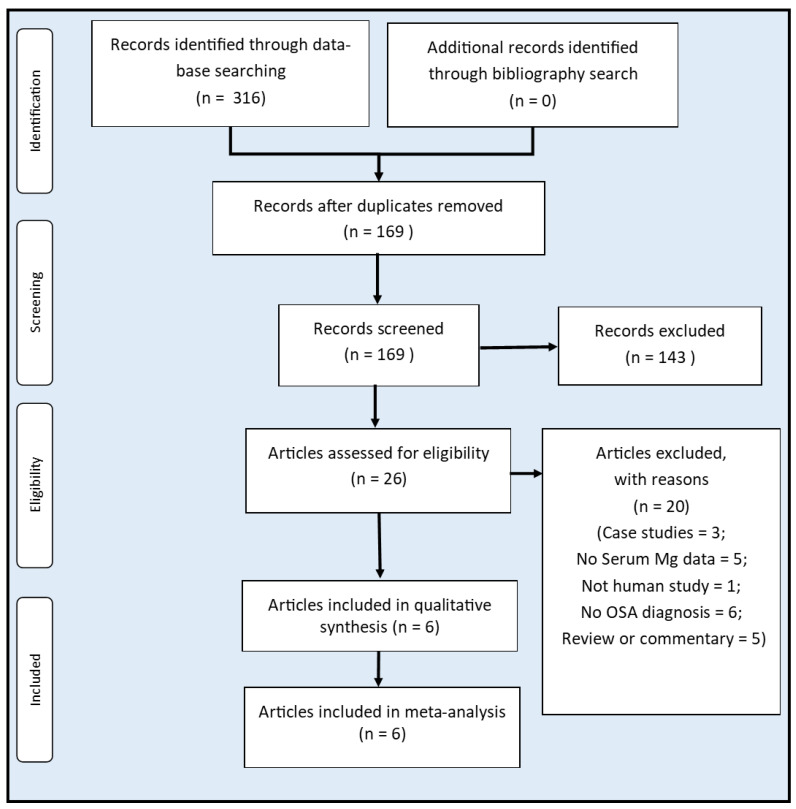
Flow diagram of study selection.

The literature search flow diagram is depicted in Figure 1. Our systematic searches yielded 169 unique records after the removal of duplicates. Following an initial screening, the full text of 26 entries was sourced and assessed for eligibility. Twenty articles were excluded with reasons (Figure 1). Six studies [21,22,23,24,25,26] are included in this systematic review and meta-analysis.

Table 1 shows a summary of the characteristics of these studies. A total of 312 OSA patients participated in these studies, with male patients out-numbering females 2.61 to 1, based on the gender split reported by five of the studies. The pooled mean age is 49.24 ± 8.94 (mean ± SD) across 279 participants (one study did not report the mean age).

### 3.2. Serum Mg and Risk of Mg Deficiency

#### 3.2.1. Comparing to Healthy Controls

Only four out of the six studies compared the serum Mg levels of OSA patients to healthy controls. At the time of diagnosis, participants in the OSA group (*n* = 68) in the study by Karamanli et al. [23] showed an average Mg level of 1.71 ± 0.21 mg/dL. These levels were significantly lower than the controls (*n* = 30) matched for age, sex and BMI, which showed healthy levels of mean serum Mg serum at 2.19 ± 0.36 mg/dL (*p* < 0.0001). These findings were similar to Xu et al. [24], where patients with OSA (*n* = 33, 1.71 ± 0.21 mg/dL) had serum Mg levels significantly lower (*p* = 0.021) than those in the control group (*n* = 33, 2.19 ± 0.36 mg/dL). Furthermore, 23 patients (69.7%) in the OSA group demonstrated hypomagnesemia.

In contrast, in the case-control study by Cakir et al. [25], there were no significant differences (*p* > 0.05) in mean serum Mg levels between the OSA group (*n* = 70, 2.0 ± 0.12 mg/dL) and healthy controls (*n* = 30, 2.04 ± 0.19 mg/dL). The participants’ characteristics in this study may have influenced the results as they were male smokers. Furthermore, seven of the OSA patients and three control subjects had diabetes, and one participant in the OSA group was hypertensive. These results were in contrast to Asker et al. [21], which evaluated serum levels of trace metals in OSA patients. The study showed that participants with OSA (*n* = 61) had significantly higher levels of trace minerals, including Mg and heavy metals, than in healthy controls (*n* = 36) (*p* < 0.001).

It is important to note that there were inconsistencies in the unit measurement of Mg in the reported findings of Asker et al. [21]. The mean for Mg for both the control group and OSA group was in micrograms per decilitre (μg/dL) (11.20 ± 7.22 and 14.13 ± 4.88, respectively). When converting these numbers to milligram per decilitre (mg/dL), the standard unit of comparison in our report, the measurements were 0.01413 mg/dL (OSA) and 0.1120 mg/dL (control), which were too low for both groups. A request for clarification to the author of this study was made. However, no response was received. The discrepancy may have resulted from errors in the recording unit of measurement. Hence, we assumed the mean Mg levels for OSA patients in this study to be 1.413 mg/dL and 1.120 mg/dL for the healthy controls, as shown in Table 1 and for the subsequent meta-analysis.

#### 3.2.2. Meta-Analysis—Effect Size

**Figure 2:**
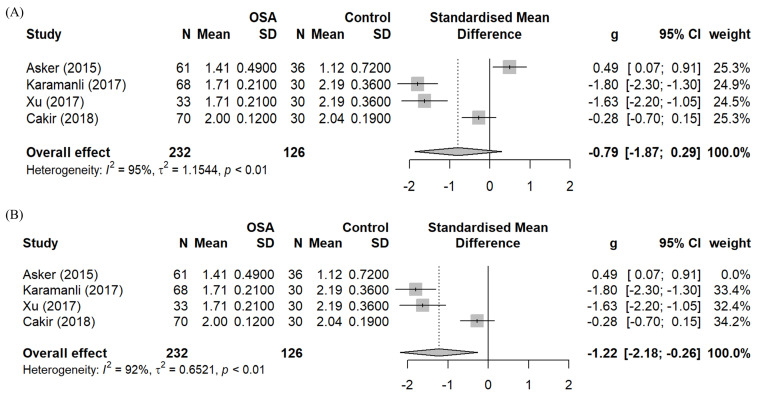
Forest plots–Meta-analyses of the standardised mean difference of the effects of OSA on serum Mg based on: (**A**) Four studies that compared the serum Mg levels of OSA patients to healthy controls [21,23,24,25]; (**B**) After exclusion of Asker et al. [21].

A meta-analysis of the effect sizes (Hedge’s g) of OSA on the serum Mg levels compared to healthy controls with data from four studies is shown as a forest plot in Figure 2a. Due to the high heterogeneity (I${}^{2}$ = 95%) a random effects model was used. The overall effect is estimated at −0.79 (95% CI: −1.87, 0.29), but it is not statistically significant.

One study, namely Asker et al. [21], skews the overall effect. The unit of measurement for Mg levels was questionable in Asker et al. [21] and the controls in this study also suffered from hypomagnesemia (1.120 mg/dL) and might not represent the healthy population. Hence, it is justifiable to eliminate this study from the meta-analysis of OSA’s effect on serum Mg levels compared to healthy control. The new analysis yields a statistically significant effect size of −1.22 (95% CI: −2.24, −0.21), as shown in Figure 2b. This analysis supports the alternate hypothesis that OSA patients’ mean serum Mg levels are lower than those of the healthy matching controls.

#### 3.2.3. Meta-Analysis—Pooled Mean

**Figure 3:**
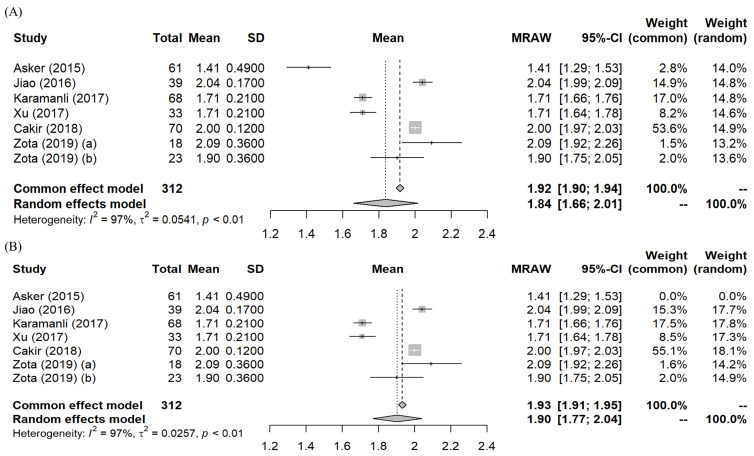
Forest plots–Meta-analyses of the mean serum Mg levels based on: (**A**) All included studies [21,22,23,24,25,26]; (**B**) After exclusion of Asker et al. [21]. (MRAW = untransformed mean). Two mean values were reported by Zota et al. [26]: (a) moderate OSA; (b) severe OSA. Both values were included in the analysis.

A meta-analysis of the mean serum Mg levels of all included studies with and without Asker et al. [21] is shown as forest plots in Figure 3. Due to the high heterogeneity (I${}^{2}$ = 97%), the random effects model results were used. The pooled means of the meta-analysis are 1.84 (95% CI: 1.66, 2.01) mg/dL with Asker et al. [21] and 1.90 (95% CI: 1.77, 2.04) mg/dL after eliminating Asker et al. [21]. An investigation of heterogeneity through systematic trial elimination was conducted. We found all studies contributed to the statistical heterogeneity since the I${}^{2}$ value did not reduce with the exclusion of any of them. Heterogeneity only reduced to moderate (I${}^{2}$ = 36%) after eliminating three studies with the lowest means [21,23,24] with participants consisting of mostly hypomagnesemia OSA patients.

The sizeable statistical heterogeneity across studies can be due to the inherently diverse characteristics of the OSA patient population. In any case, the pooled means of either 1.84 (95% CI: 1.66, 2.01) mg/dL or 1.90 mg/dL (95% CI: 1.77, 2.04) do not differ significantly from the reference range of normal healthy population (mean = 2.07 mg/dL, 95% CI: 1.82, 2.30). Hence, results from the present analysis do not support the hypothesis that OSA patients have lower than normal serum Mg levels in general. However, with the 95% CI of both pooled means below the healthy cut-off value of 2.07 mg/dL as suggested by Costello et al. [11], we can conclude these patients are at risk of Mg deficiency.

### 3.3. Serum Mg and OSA Severity

#### 3.3.1. AHI

When comparing patients with different OSA severities, Zota et al. [26] showed that those with severe OSA (AHI ≥ 30, *n* = 23) had reduced serum Mg levels when compared to those with moderate OSA (15 ≤ AHI < 30, *n* = 18), but the mean difference (MD) was not statistically significant (MD = 0.19, *p* = 0.1). It is worth noting that comorbidities of hypertension, impaired fasting glucose, and dyslipidemia were common among moderate to severe OSA patients recruited in this study. Similarly, Karamanli et al. [23] reported no significant difference in serum Mg between patients with moderate OSA and those with severe symptoms. However, the mean serum Mg level of patients with mild OSA was significantly higher than those in the severe group (1.84 ± 0.1 vs. 1.67 ± 0.26; *p* = 0.003) [23]. Additionally, in multiple regression analysis, Karamanli et al. [23] found that the serum Mg level was still significantly associated with AHI (β = −0.03, *p* = 0.01) after adjusting for age, gender, and BMI.

#### 3.3.2. Before and after Treatment

The severity of OSA symptoms generally improved after therapy. Two studies measured the serum Mg of OSA patients before and after treatment. In an observation study by Jiao et al. [22], postoperative blood Mg levels significantly increased in OSA patients (*n* = 39), six to 12 months after Roux-en-Y gastric bypass (RYGB) for weight loss, compared to pre-intervention levels (2.04 ± 0.17 mg/dL vs. 2.19 ± 0.17 mg/dL, *p* < 0.05). The RYGB surgery also improved AHI severity ratings with an MD of 12.64 (95% CI: −16.81, −8.4; *p* < 0.001). However, the improvement in AHI appeared uncorrelated with serum Mg based on Pearson and Spearman correlation analysis. This study, however, suffered from a high drop-out rate, with 15 out of the initial 54 recruits (27.7%) not completing the study.

In the cross-sectional study of Xu et al. [24], 22 out of the 33 OSA patients were followed up after three months of continuous positive airway pressure (CPAP) treatment. An improvement in serum Mg levels was observed with a mean of 2.02 ± 0.25 mg/dL, which was significantly higher (*p* < 0.001) than the baseline value of 1.71 ± 0.21 mg/dL. Pearson correlation between serum Mg and AHI was almost statistically significant (r = −0.302, *p* = 0.056). Nevertheless, their post-CPAP mean serum Mg remained significantly lower than the control group’s baseline value (vs. 2.19 ± 0.36 mg/dL; *p* = 0.05). In multivariate logistic regression analysis, serum Mg levels ≥ 1.98 mg/dL were shown to be a protective factor for OSA severity, with participants being less likely to have severe OSA than those with serum Mg level below 1.98 mg/dL (Odds Ratio = 0.54, 95% CI: 0.38, 0.88; *p* = 0.006) [24].

### 3.4. Serum Mg and Biomarkers

#### 3.4.1. Lipid Profile

Two studies reported the correlation between serum Mg levels and lipid metabolism indices. Using Spearman’s rho tests, Asker et al. [21] showed that serum Mg was positively correlated with triglycerides (r = 0.340, *p* = 0.003), negatively correlated with high-density lipoproteins (HDL) (r = −0.244, *p* = 0.003), while correlations with total cholesterol and low-density lipoproteins (LDL) were not significant. Çakır et al. [25], on the other hand, did not detect any statistically significant Spearman correlations between serum Mg and any of the lipid biomarkers.

#### 3.4.2. Glucose Metabolism

Jiao et al. [22] reported that serum Mg levels appeared to increase along with improved blood glucose control after RYGB. However, no significant correlation was found between the AHI and fasting glucose and insulin before and after RYGB surgery. In contrast, Çakır et al. [25] found evidence that supported the association between serum Mg and glucose metabolism biomarkers, which include fasting serum glucose, serum fasting insulin, and homeostatic model assessment-insulin resistance. These three biomarkers were significantly different between OSA patients and controls (*p* = 0.004, *p* = 0.003, *p* < 0.001, respectively). Meanwhile, only fasting serum glucose had a significantly negative correlation with serum Mg (Spearman’s r = −0.384, *p* = 0.001). Similarly, these biomarkers were significantly higher (*p* = 0.033, *p* = 0.036, *p* = 0.003, respectively) in patients with severe OSA than those with only mild/moderate symptoms. On the contrary, Zota et al. [26] reported no significant difference in fasting serum glucose between severe and moderate OSA groups.

#### 3.4.3. Trace Minerals and Heavy Metals

In addition to Mg, Asker et al. [21] also assessed the serum levels of trace minerals (copper, iron, zinc, manganese, and cobalt) and heavy metals (lead and cadmium) in both OSA patients and non-apnoeic controls. The OSA group showed significantly higher levels of all these minerals (*p* = 0.002 for manganese and *p* < 0.001 for the rest) except cadmium. Mg had significantly positive Spearman’s correlations (*p* < 0.001) with these trace minerals while correlating negatively with lead. Since Mg is a physiological calcium (Ca) antagonist, Çakır et al. [25] hypothesised that the Ca/Mg ratio could be more informative than evaluating Mg or Ca alone in OSA patients. Their study found that, even though OSA patients had numerically higher Ca/Mg ratios than healthy controls, the difference is insignificant. In a subgroup analysis, the Ca/Mg ratios were compared between the severe OSA patients and mild/moderate OSA patients, and the difference was statistically significant (4.83 ± 0.48 vs. 4.51 ± 0.39, *p* = 0.17).

#### 3.4.4. C-Reactive Protein, Ischemia-Modified Albumin, and Carotid Intima-Media Thickness

Karamanli et al. [23] evaluated the relationship between serum Mg levels and the inflammatory response in patients with OSA. In addition to showing that Mg levels were lower in patients with OSA, the study also showed that patients with OSA had substantially higher plasma C-reactive protein (CRP) concentrations than controls (7.6 ± 1.3 g/dL in OSA group with *n* = 68, 2.7 ± 1.5 g/dL in control group with *n* = 30, *p* < 0.0001). The study showed a significant difference in the mean Mg and CRP levels based on OSA severity (AHI scores of 5–15 vs. ≥30) but independent of BMI (*p* = 0.001). Hence, serum Mg levels reduced depending on the presence of OSA and its severity, and low Mg levels are associated with higher levels of CRP concentrations.

Xu et al. [24] investigated whether the analysis of Mg, high sensitivity C-reactive protein (hsCRP), and ischemia-modified albumin (IMA) concentrations can be used as a method of diagnosis for OSA. The study showed that patients with OSA (*n* = 33) had higher hsCRP concentrations than those without (*n* = 30) (1.47 ± 1.60 mg/L vs. 0.97 ± 1.22 mg/L; *p* < 0.05). The study also showed that there was a significant correlation between serum IMA (r = 0.614; *p* < 0.001) and hsCRP (r = −0.453; *p* < 0.001) levels and AHI concluding that patients with OSA have reduced Mg levels and higher serum hsCRP and IMA levels. Serum IMA also occurred at higher levels in participants with OSA than in the control group (0.43 ± 0.09 absorbance units, *p* < 0.05). These levels, however, were reversed by CPAP treatment interventions. It should be noted that CPAP is known to have a high non-adherence rate with many patients eventually abandoning the device. Xu et al. [24] did not track and report the adherence data of CPAP treatment, which represents a major drawback of this study and casts doubts on the validity of their findings.

Asker et al. [21] also assessed CRP levels based on OSA diagnosis; the study showed that patients with OSA (*n* = 61) had higher levels of CRP compared to control groups (*n* = 36) (severe OSA: 0.52 mg/dL vs. control: 0.40mg/dL). The study showed that carotid intima-media thickness (CIMT), the test used to measure the carotid artery’s inner two layers, was increased in OSA patients. This increase in CIMT was correlated with levels of cobalt, copper, iron, Mg, and manganese.

### 3.5. Quality Assessment

**Figure 4:**
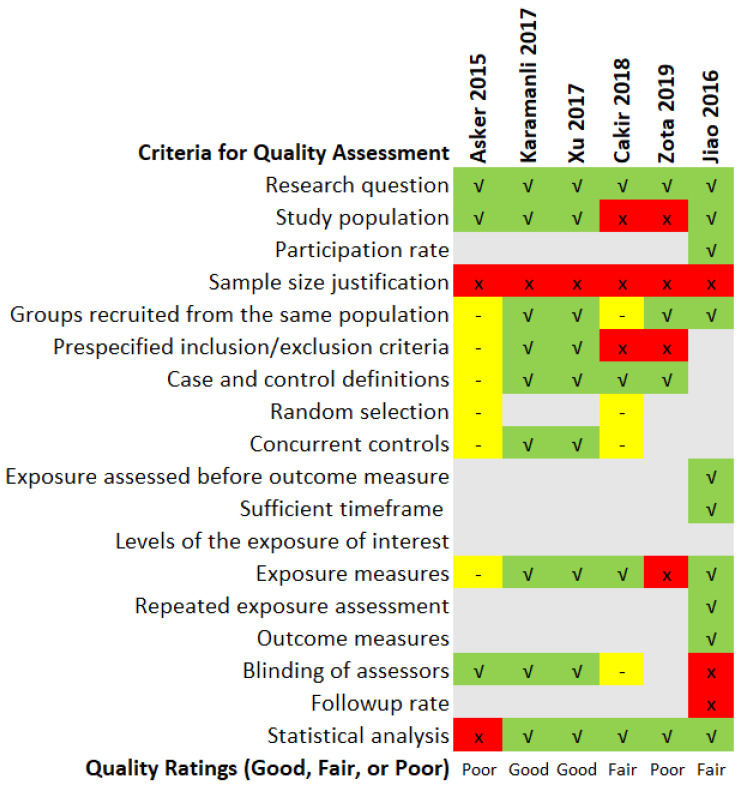
Quality assessment ratings of the included studies [21,22,23,24,25,26]. Legend: Green: Yes; Red: No; Gray: Not Applicable; Yellow: Not Reported or Cannot Determine.

The quality assessment ratings and findings of the six included articles are summarised in Figure 4. Two studies were rated as poor quality, of which the poor rating of Asker et al. [21] was due to discrepancies in serum Mg unit reporting mentioned earlier. The study by Zota et al. [26] was also deemed unsatisfactory due to the ambiguity in the study population selection criteria. The report also offers no details for the length of the study and when the study was carried out. The diagnostic method of OSA also varied between participants as some were diagnosed using take-home sleep tests and others based on hospital overnight sleep tests. Studies such as Cakir et al. [25] and Jiao et al. [22] were rated as fair as they had some unreported gaps such as justification in the sample size of participants, blinding of accessors or had low follow-up rates. A more detailed version of the quality assessment rating chart is available in the Supplementary Materials section.

## 4. Discussion

Based on our findings, there is evidence suggesting that: (1) OSA patients have lower serum Mg levels than healthy controls, even though their serum Mg levels may still fall within the normal reference range. (2) OSA patients are at risk of Mg deficiency. (3) OSA severity appears to affect the serum Mg levels; the higher the AHI, the lower the serum Mg. (4) Serum Mg levels of OSA patients correlate with biomarkers including CRP, IMA and CIMT for cardiovascular disease risks. The serum Mg levels also potentially correlate with biomarkers for lipid profile, glucose metabolism, Ca and heavy metals.

Our results were consistent with studies that displayed serum Mg levels correlating with other metabolic, endocrine and cardiovascular disorders [27,28,29]. However, the direct relationship between these conditions, OSA, and serum Mg levels is complex and multifactorial. It is still unclear whether OSA directly causes a lower serum Mg level or whether the lower serum Mg level is one of the risk factors in OSA. Factors suggested to impact serum Mg levels in OSA patients include oxidative stress, insufficient dietary intake, and impaired Mg regulation due to other comorbidities.

Sleep deprivation is a form of stress and can alter behavioural, physiological, and cellular functioning. In an animal study investigating the effect of Mg and sleep deprivation, Akanmu et al. [30] showed that sleep deprivation significantly (*p* < 0.05) decreased the plasma levels of free Mg${}^{2+}$ and Ca${}^{2+}$. The study also identified sleep deprivation as a contributing factor to the loss of Mg and Ca electrolytes and the problems associated with the integrity of physiological system functions. The loss of electrolytes can affect motor function as it was observed in a human study that chronic sleep deprivation reduced intracellular Mg and decreased exercise tolerance, which was subsequently corrected with oral Mg administration [31]. Furthermore, intermittent nocturnal hypoxia was also significantly associated with oxidative stress, increased pro-inflammatory markers, and OSA, according to Orrù et al. [32]. Similarly, correcting low Mg status with supplementation has also been shown to improve inflammatory and oxidative stress markers in adults experiencing poor sleep quality, according to a placebo-controlled study by Nelson et al. [33].

In a recent clinical study of patients with type 2 diabetes, a condition often associated with OSA, hypomagnesaemia was seen to be a significant pathogenic factor that causes increased oxidative stress. Mg deficiency was seen to enhance oxidate stress marker and was shown to have a significant negative correlation with serum malondialdehyde, an indicator of oxidative stress [34]. Other studies have suggested that disturbances of trace mineral metabolism were due to oxidative stress and inflammatory response. OSA affects the absorption and circulating levels of these substances [35,36,37]. Increased intestinal absorption or release during tissue damage may also contribute to the increased serum levels of trace minerals [38,39]. If the balance is disturbed, excessive trace minerals may induce oxidative stress, leading to a vicious circle that reinforces chronic inflammation. Elimination of heavy metals may not be achieved due to oxidative stress and inflammation, and this may explain the high levels of cadmium and lead in the setting of OSA [21]. Lead is a pro-inflammatory heavy metal that helps amplify oxidative stress and interferes with divalent cations such as Mg and may lead to reduced levels of serum Mg. Asker et al. [21] showed that OSA patients had higher levels of heavy metals and that Mg negatively correlated with lead. As Mg is a cofactor in more than 325 enzymes in the human body, disorders associated with Mg availability or function have been suggested to promote oxidative stress [40]. In OSA, nocturnal hypoxia was significantly associated with oxidative stress and the increase in pro-inflammatory markers [32]. This complex relationship between trace mineral metabolism, oxidative stress, and Mg levels in OSA warrants further investigation.

Mechanisms associated with impaired Mg regulation can be related to common diseases, which can be affected by dietary intake. Dietary analysis of 93 OSA patients by Bronkowska et al. [41] reported a low intake of high Mg foods. The patients had a mean Mg intake of only 194.0 mg and did not meet the recommended dietary allowance of 280.0 mg. The participants had slight deficiencies in Mg and other minerals such as calcium, zinc and iron. Notably, in this study, 75% of women and 91.9% of men received CPAP treatment which could help to increase Mg levels. Thus, the serum Mg levels in these patients could be lower without intervention. Cao et al. [42] investigated Mg intake and sleep disorder symptoms in adults (*n* = 1487) using a food diary and sleep quality questionnaires. The study showed no associations between dietary Mg intake and daytime sleepiness or night snoring in either gender. The study concluded that Mg supplementation might have long-term benefits in reducing the likelihood of daytime falling asleep in women. The lack of clinical diagnosis of sleep disorders and serum analysis of potential nutritional deficiencies in this study does impact the validity of these results and conclusions. Controlled, clinical-based trials on the effect of Mg intake and sleep disorder conditions such as OSA can help investigate these links for more accurate findings.

A growing body of literature has shown a pathological role for Mg deficiency. Ismail et al. [29] reviewed 221 peer-reviewed studies published from 1990 to April 2015 and found the association of Mg deficiency with increased risk and prevalence in multiple conditions, including cardiovascular pathology, electrolyte disorders, hypertension, endocrine and metabolic disorders, muscular, neurological disorders and even some cancers such as colorectal cancer. This inverse relationship was seen irrespective of the methods used to assess Mg body stores. Similarly, the review found 79 studies where Mg deficiency was found to predict adverse events, and a reduced risk of pathology was seen when supplementation was introduced.

**Figure 5:**
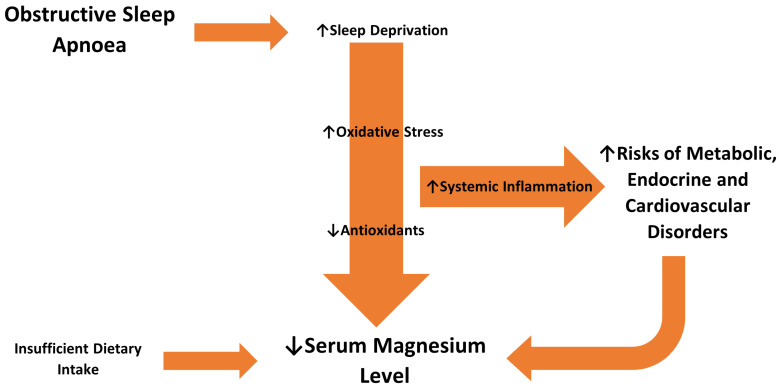
Hypothesis: OSA as a causative factor in lowering serum Mg level.

Based on our findings and the evidence in the literature discussed above, we deduce that OSA is a causative factor in the observed lower serum Mg level. OSA leads to sleep deprivation and thus increases oxidative stress. The increased oxidative stress demands Mg, a cofactor of several antioxidant enzymes, including superoxide dismutase. The depletion of antioxidants in the body disrupts the Mg balance. It can also promote chronic systemic inflammation, which increases the risks of other metabolic, endocrine and cardiovascular disorders, impairing Mg regulation. The diminishing Mg level can be further compounded by insufficient dietary intake. This cascade of events is shown in Figure 5. Such a hypothesis will need to be validated in future research.

As with all research, this review has limitations. The strict inclusion criteria used in the meta-analysis conducted allowed for a small number of studies. They are not likely to represent OSA patients in general. Furthermore, with so few studies, the validity of the meta-analysis on serum mg levels in OSA patients as reported in this study remains questionable. The secondary qualitative analyses on OSA’s correlations with other metabolic, nutritional, inflammatory, or cardiac markers have relied on even few studies with contrasting results. Hence, the findings are far from definitive and must be interpreted with caution. Moreover, the review included only publications in English, and there may be findings from other languages that we did not include. An epidemiology study investigating the serum Mg levels in OSA patients is warranted. Such a study can help provide more accurate data for drawing definitive conclusions.

Notwithstanding its limitations, this study is a novel attempt to systematically review and analyse an underrepresented research area. Our research has identified a gap in understanding the correlation between Mg serum levels and OSA. There are also insufficient studies investigating the definitive role and use of Mg supplements in OSA management. Further research investigating the link between Mg levels, OSA, and systemic inflammatory markers is worthwhile as correcting Mg deficiency through diet or supplementation can be an affordable treatment option addressing both OSA and its chronic comorbidities.

## 5. Conclusions

The link between Mg and OSA remains poorly understood, with some studies showing a potentially significant relationship between OSA severity and serum Mg levels. This study is the first systematic review and meta-analysis of its kind, and it provides evidence that OSA patients are at risk of Mg deficiency. An improvement in serum Mg levels of OSA patients after intervention presents a promising area of research for the overall management of both Mg deficiency and OSA. More research, public health awareness, and emphasis on the importance of dietary Mg are needed for all age groups to help reduce its potential insufficiency and the associated risks.

## Figures and Tables

**Table 1:** Characteristics of the included studies.

Study and Design	Participants	Objective	Findings: Mean Serum Mg (mg/dL) and Metabolic Parameters	Ref.
Asker (2015) Turkey Cross-sectional case-control study	OSA: AHI ≥ 30, *n* = 61. Male/Female: 42/19. Mean age: 48.2 ± 9.1. Control: healthy volunteers, *n* = 36. Male/Female: 11/25. Mean age: 46.9 ± 10.2.	Evaluation of serum levels of trace minerals and heavy metals in severe OSA patients before any therapeutic intervention.	OSA: 1.413 ± 0.488; Control: 1.12 ± 0.722; OSA > Control (*p* < 0.001). The OSA group had significantly higher cholesterol, triglycerides, trace minerals, heavy minerals, and CIMT compared to the control (*p* < 0.005 for all). CIMT and triglycerides were positively correlated with Mg. HDL was negatively correlated with Mg.	[21]
Jiao (2016) China Case-control follow up observational study	OSA patients (AHI ≥ 5) with obesity and T2DM receiving RYGB surgery, *n* = 39. Male/Female: 15/24. Mean age: 44.20 ± 8.95 (Males); 50.50 ± 12.29 (Females).	Assessing the efficacy of RYGB surgery on patients with OSA using PSG and biochemical tests (including Mg levels), pre- and post-surgery. Study duration: 6–12 months post-intervention.	Preoperative: 2.04 ± 0.17; Postoperative: 2.19 ± 0.17; Post > Pre (*p* < 0.001). RYGB surgery significantly lowered the AHI and BMI in OSA patients. Postoperative blood Mg levels were significantly increased in OSA patients when compared to the time of diagnosis (*p* < 0.05). Mg was not correlated with the improvement in AHI.	[22]
Karamanli (2017) Turkey Retrospective cross-sectional study	OSA: AHI ≥ 5, *n* = 68. Male/Female: 46/22. Control: healthy volunteers, *n* = 30. Male/Female: 14/16.	To evaluate the relationship between serum levels of Mg and the inflammatory response (CRP) in patients with newly diagnosed OSA.	OSA: 1.71 ± 0.21; Control: 2.19 ± 0.36; OSA < Control (*p* < 0.0001). Mg levels were lower in OSA patients than those in controls. Those with severe OSA also had significantly lower Mg (*p* = 0.03) than those with mild OSA. OSA group had a significantly higher CRP. A significant negative correlation was observed between Mg and CRP levels (*p* < 0.0001).	[23]
Xu (2017) China Case-control study with subgroup follow-up	OSA: AHI ≥ 5, *n* = 33. Male/Female: 23/10. Mean age: 51.6 ± 9.8. Control: healthy volunteers, *n* = 30. Male/Female: 21/9. Mean age: 52.1 ± 10.9.	Investigating Mg, hsCRP, and IMA as a non-invasive diagnosis method for OSA. Participants followed up after 3 months of CPAP treatment.	OSA: 1.71 ± 0.21; Control: 2.19 ± 0.36; OSA < Control (*p* = 0.021). Post-CPAP: 2.02 ± 0.25 (*n* = 22 only); Post > Baseline (*p* < 0.001) OSA patients had significantly higher levels of hsCRP and IMA and significantly lower Mg compared to control (*p* < 0.05 for all). Mg, hsCRP, and IMA were used in a novel model to diagnose OSA with AUROC of 0.93 (0.83–0.98). OSA patients showed significant improvements in Mg, hsCRP, and IMA after CPAP treatment.	[24]
Cakir (2018) Turkey Clinical case-control study	OSA: AHI ≥ 5, *n* = 70 (55 severe, 11 moderate and 4 mild). Male only. Mean age: 47.57 ± 12.15. Control: non-apnoeic individuals (AHI < 5), *n* = 30. Male only. Mean age: 43.23 ± 10.5.	Comparing OSA patients’ metabolic markers (BMI, PSG, insulin sensitivity–resistance markers, lipid profiles) and mineral levels with those of control subjects.	OSA: 2.0 ± 0.12; Control: 2.04 ± 0.19. There were no statistically significant differences in serum Mg, Ca, and Ca/Mg ratios between OSA patients and controls. Fasting glucose and insulin levels were significantly higher in the OSA group (*p* < 0.05 for all). Fasting glucose levels were correlated with Ca, Mg, and Ca/Mg ratios. Severe OSA patients had significantly higher Ca/Mg ratios than mild/moderate groups (*p* = 0.017).	[25]
Zota (2019) Romania Cross-sectional, single cohort study	OSA: AHI ≥ 5, *n* = 41 (23 severe and 18 moderate/mild). Mean age: 55.83 (moderate/mild), 57.34 (severe).	Assessed the relationship between OSA severity, arterial stiffness and clinic-biological parameters in moderate-severe OSA patients prior to the use of CPAP therapy.	Moderate: 2.09; Severe: 1.9; SD not reported. Between-group Δ not significant with *p* = 0.1. No significant difference was found between severe and moderate groups in Mg levels. ESR was significantly higher in the severe group (*p* = 0.012). Inflammation markers (CRP and ESR) were correlated with OSA severity. Mg was negatively correlated with PWV, which measured arterial stiffness.	[26]

Abbreviations: AHI, apnoea-hypopnoea index; AUROC, area under the receiver operating characteristics; BMI, body mass index; Ca, calcium; CPAP, continuous positive airway pressure; CRP, C-reactive protein; CIMT, carotid intima-media thickness; ESR, erythrocyte sedimentation rate; HDL, high-density lipoproteins; hsCRP, high sensitivity CRP; IMA, ischemia-modified albumin; Mg, magnesium; OSA, obstructive sleep apnoea; PSG, polysomnography; PWV, pulse wave velocity; RYGB, Roux-en-Y gastric bypass; T2DM, type 2 diabetes mellitus; SD, standard deviation.

## Data Availability

Meta-analysis data are available as HTML outputs from R Studio, downloadable from the Supplementary Materials section above.

## Supplementary Materials

The following supporting information can be downloaded at: https://www.mdpi.com/article/10.3390/biomedicines10092273/s1, (1) Figure S1: A detail quality assessment rating chart; (2) The PRISMA 2020 Checklist; (3) Html outputs from R Studio for the meta-analysis. Reference [43] is cited in the supplementary materials.

## Abbreviations

The following abbreviations are used in this manuscript:

AHI	Apnoea-hypopnoea index
AUROC	Area under the receiver operating characteristics
BMI	Body mass index
Ca	Calcium
CPAP	Continuous positive airway pressure
CRP	C-reactive protein
CI	Confidence interval
CIMT	Carotid intima-media thickness
ESR	Erythrocyte sedimentation rate
HDL	High-density lipoproteins
hsCRP	high sensitivity CRP
IMA	Ischemia-modified albumin
LDL	Low-density lipoproteins
Mg	Magnesium
N	Sample size
OSA	Obstructive sleep apnoea
PSG	Polysomnography
PWV	Pulse wave velocity
RYGB	Roux-en-Y gastric bypass
T2DM	Type 2 diabetes mellitus
SD	Standard deviation
