# Supplementary material for: Serum Magnesium Levels in Patients with Obstructive Sleep Apnoea: A Systematic Review and Meta-Analysis
Source: Biomedicines. 2022 Sep 14;10(9):2273. doi: 10.3390/biomedicines10092273 (PMC9496273; doi:10.3390/biomedicines10092273)
Supplement: Supplementary file 1 [file biomedicines-10-02273-s001.zip › FigureS1.pdf]

Figure S1. Quality assessment ratings

Criteria for Quality Assessment for Case-Control Studies	Asker et al. 2015			Karamanli et al. 2017			Xu et al. 2017			Cakir et al. 2018			Zota et al. 2019			Criteria for Quality Assessment for Observational Cohort and Cross-Sectional Studies	Jaio et al. 2016		
	Yes	No	Other (CD, NR, NA)	Yes	No	Other (CD, NR, NA)	Yes	No	Other (CD, NR, NA)	Yes	No	Other (CD, NR, NA)	Yes	No	Other (CD, NR, NA)		Yes	No	Other (CD, NR, NA)
1. Was the research question or objective in this paper clearly stated and appropriate?	√			√			√			√			√			1. Was the research question or objective in this paper clearly stated?	√		
2. Was the study population clearly specified and defined?	√			√			√				√			√		2. Was the study population clearly specified and defined?	√		
3. Did the authors include a sample size justification?		√			√			√			√			√		3. Was the participation rate of eligible persons at least 50%?	√		
4. Were controls selected or recruited from the same or similar population that gave rise to the cases (including the same timeframe)?			NR	√			√					NR	√			4. Were all the subjects selected or recruited from the same or similar populations (including the same time period)? Were inclusion and exclusion criteria for being in the study prespecified and applied uniformly to all participants?	√		
5. Were the definitions, inclusion and exclusion criteria, algorithms or processes used to identify or select cases and controls valid, reliable, and implemented consistently across all study participants?			CD	√			√				√			√		5. Was a sample size justification, power description, or variance and effect estimates provided?		√	
6. Were the cases clearly defined and differentiated from controls?			CD	√			√			√			√			6. For the analyses in this paper, were the exposure(s) of interest measured prior to the outcome(s) being measured?	√		
7. If less than 100 percent of eligible cases and/or controls were selected for the study, were the cases and/or controls randomly selected from those eligible?			NR			NA			NA			NR			NR	7. Was the timeframe sufficient so that one could reasonably expect to see an association between exposure and outcome if it existed?	√		
8. Was there use of concurrent controls?			CD	√			√					CD			CD	8. For exposures that can vary in amount or level, did the study examine different levels of the exposure as related to the outcome (e.g., categories of exposure, or exposure measured as continuous variable)?			NA
9. Were the investigators able to confirm that the exposure/risk occurred prior to the development of the condition or event that defined a participant as a case?			NA			NA			NA			NA			NA	9. Were the exposure measures (independent variables) clearly defined, valid, reliable, and implemented consistently across all study participants?	√		
10. Were the measures of exposure/risk clearly defined, valid, reliable, and implemented consistently (including the same time period) across all study participants?			CD	√			√			√				√		10. Was the exposure(s) assessed more than once over time?	√		
11. Were the assessors of exposure/risk blinded to the case or control status of participants? (subjective assessment) – radio.	√			√			√					NR			NR	11. Were the outcome measures (dependent variables) clearly defined, valid, reliable, and implemented consistently across all study participants?	√		
12. Were key potential confounding variables measured and adjusted statistically in the analyses? If matching was used, did the investigators account for matching during study analysis?		√		√			√			√			√			12. Were the outcome assessors blinded to the exposure status of participants?		√	
																13. Was loss to follow-up after baseline 20% or less?		√	
																14. Were key potential confounding variables measured and adjusted statistically for their impact on the relationship between exposure(s) and outcome(s)?	√		
**Quality Rating (Good, Fair, or Poor)**	Poor			Good			Good			Fair			Poor			**Quality Rating (Good, Fair, or Poor)**	Fair		
**Additional Comments**	Unreliable report of variables – were inconsistent in report; Mg levels reports varied in study figures.									The study population and time period not clearly specified. Some unreported gaps including no justification of sample size of participants and whether assessors were blind to participant information.			The study population and time period was not clearly specified. Participation rate of eligible persons not reported. Exclusion/inclusion criteria not specified. Method of OSA diagnosis varied between participants.			**Additional Comments**	Study had low follow up rates – over 20% loss		
