# Supplementary material for: Serum Magnesium Levels in Patients with Obstructive Sleep Apnoea: A Systematic Review and Meta-Analysis
Source: Biomedicines. 2022 Sep 14;10(9):2273. doi: 10.3390/biomedicines10092273 (PMC9496273; doi:10.3390/biomedicines10092273)
Supplement: Supplementary file 1 [file biomedicines-10-02273-s001.zip › metaanalyisis_means.html]

Meta-analyses of the mean serum Mg levels of included studies


# Meta-analyses of the mean serum Mg levels of included studies

## Setup

### Load packages

```
library(reshape2)
library(ggplot2)
library(dplyr)
library(statsr)
library(meta)
library(metafor)
library(units)
```

### Load data

```
# Load and display the data
rawdata<-read.csv("./data2021.csv", header=TRUE)
rawdata
```

```
##              study  n male female age_mean age_sd mg_mean mg_sd
## 1     Asker (2015) 61   42     19    48.20   9.10    1.41  0.49
## 2      Jiao (2016) 39   15     24    49.57  11.03    2.04  0.17
## 3 Karamanli (2017) 68   46     22    47.20   1.20    1.71  0.21
## 4        Xu (2017) 33   23     10       NA     NA    1.71  0.21
## 5     Cakir (2018) 70   70      0    47.57  12.15    2.00  0.12
## 6  Zota (2019) (a) 18   NA     NA    55.83   7.05    2.09  0.36
## 7  Zota (2019) (b) 23   NA     NA    57.34   7.05    1.90  0.36
```

```
attach(rawdata)
```

```
# Use metamean to pool the mean from all studies
m1 <- meta::metamean(n,mg_mean,mg_sd, studlab = study, title = "Meta Analysis of Serum Mg Levels in OSA patients")

m1
```

```
## Review:     Meta Analysis of Serum Mg Levels in OSA patients
## 
## Number of studies combined: k = 7
## Number of observations: o = 312
## 
##                        mean           95%-CI
## Common effect model  1.9156 [1.8951; 1.9362]
## Random effects model 1.8363 [1.6598; 2.0129]
## 
## Quantifying heterogeneity:
##  tau^2 = 0.0541 [0.0208; 0.2795]; tau = 0.2326 [0.1443; 0.5287]
##  I^2 = 97.3% [96.0%; 98.2%]; H = 6.08 [4.98; 7.42]
## 
## Test of heterogeneity:
##       Q d.f.  p-value
##  221.54    6 < 0.0001
## 
## Details on meta-analytical method:
## - Inverse variance method
## - Restricted maximum-likelihood estimator for tau^2
## - Q-Profile method for confidence interval of tau^2 and tau
## - Untransformed (raw) means
```

```
meta::forest.meta(m1, comb.fixed=FALSE, xlim= c(1.2,2.5), plotwidth= unit(8, "cm"), colgap=unit(2, "mm"), prediction = FALSE)
```

```
m2 <- meta::metamean(n,mg_mean,mg_sd, exclude = c(1), studlab = study, title = "Meta Analysis of Serum Mg Levels in OSA patients")

m2
```

```
## Review:     Meta Analysis of Serum Mg Levels in OSA patients
## 
## Number of studies combined: k = 6
## Number of observations: o = 312
## 
##                        mean           95%-CI
## Common effect model  1.9302 [1.9093; 1.9511]
## Random effects model 1.9032 [1.7691; 2.0373]
## 
## Quantifying heterogeneity:
##  tau^2 = 0.0257 [0.0088; 0.1631]; tau = 0.1603 [0.0937; 0.4038]
##  I^2 = 96.8% [94.9%; 98.0%]; H = 5.56 [4.42; 7.00]
## 
## Test of heterogeneity:
##       Q d.f.  p-value
##  154.71    5 < 0.0001
## 
## Details on meta-analytical method:
## - Inverse variance method
## - Restricted maximum-likelihood estimator for tau^2
## - Q-Profile method for confidence interval of tau^2 and tau
## - Untransformed (raw) means
```

```
meta::forest.meta(m2, xlim= c(1.2,2.5), plotwidth= unit(8, "cm"), colgap=unit(2, "mm"), comb.fixed = FALSE)
```

```
# m1, m2: the sample means
# s1, s2: the sample standard deviations
# n1, n2: the same sizes
# m0: the null value for the difference in means to be tested for. Default is 0. 
# equal.variance: whether or not to assume equal variance. Default is FALSE. 
t.test2 <- function(m1,m2,s1,s2,n1,n2,m0=0,equal.variance=FALSE)
{
    if( equal.variance==FALSE ) 
    {
        se <- sqrt( (s1^2/n1) + (s2^2/n2) )
        # welch-satterthwaite df
        df <- ( (s1^2/n1 + s2^2/n2)^2 )/( (s1^2/n1)^2/(n1-1) + (s2^2/n2)^2/(n2-1) )
    } else
    {
        # pooled standard deviation, scaled by the sample sizes
        se <- sqrt( (1/n1 + 1/n2) * ((n1-1)*s1^2 + (n2-1)*s2^2)/(n1+n2-2) ) 
        df <- n1+n2-2
    }      
    t <- (m1-m2-m0)/se 
    dat <- c(m1-m2, se, t, 2*pt(-abs(t),df))    
    names(dat) <- c("Difference of means", "Std Error", "t", "p-value")
    return(dat) 
}

t.test2(1.84, 2.07, 1.30674,15.40134,312, 15820)
```

```
## Difference of means           Std Error                   t             p-value 
##          -0.2300000           0.1430620          -1.6076945           0.1079854
```

```
t.test2(1.90, 2.07, 1.09,15.40134, 251 ,15820)
```

```
## Difference of means           Std Error                   t             p-value 
##          -0.1700000           0.1404536          -1.2103638           0.2262157
```
