# Supplementary material for: Serum Magnesium Levels in Patients with Obstructive Sleep Apnoea: A Systematic Review and Meta-Analysis
Source: Biomedicines. 2022 Sep 14;10(9):2273. doi: 10.3390/biomedicines10092273 (PMC9496273; doi:10.3390/biomedicines10092273)
Supplement: Supplementary file 1 [file biomedicines-10-02273-s001.zip › metaanalysis_effects.html]

Meta-analyses of the standardised mean difference of the effects of OSA on serum Mg


# Meta-analyses of the standardised mean difference of the effects of OSA on serum Mg

## Setup

### Load packages

```
library(reshape2)
library(ggplot2)
library(dplyr)
library(statsr)
library(meta)
library(metafor)
library(units)
```

### Load data

```
# Load and display the data
rawdata<-read.csv("./DataMD.csv", header=TRUE)
rawdata
```

```
##              Study   Me   Se   Mc   Sc Ne Nc
## 1     Asker (2015) 1.41 0.49 1.12 0.72 61 36
## 2 Karamanli (2017) 1.71 0.21 2.19 0.36 68 30
## 3        Xu (2017) 1.71 0.21 2.19 0.36 33 30
## 4     Cakir (2018) 2.00 0.12 2.04 0.19 70 30
```

```
attach(rawdata)
str(rawdata)
```

```
## 'data.frame':    4 obs. of  7 variables:
##  $ Study: chr  "Asker (2015)" "Karamanli (2017)" "Xu (2017)" "Cakir (2018)"
##  $ Me   : num  1.41 1.71 1.71 2
##  $ Se   : num  0.49 0.21 0.21 0.12
##  $ Mc   : num  1.12 2.19 2.19 2.04
##  $ Sc   : num  0.72 0.36 0.36 0.19
##  $ Ne   : int  61 68 33 70
##  $ Nc   : int  36 30 30 30
```

```
#Fixed Effect
rm.raw <- metacont(Ne,
                  Me,
                  Se,
                  Nc,
                  Mc,
                  Sc,
                  data=rawdata,
                  studlab=paste(Study),
                  comb.fixed = TRUE,
                  comb.random = FALSE,
                  prediction=TRUE,
                  sm="SMD")
rm.raw
```

```
## Number of studies combined: k = 4
## Number of observations: o = 358
## 
##                         SMD             95%-CI     z  p-value
## Common effect model -0.5941 [-0.8287; -0.3595] -4.96 < 0.0001
## Prediction interval         [-5.9883;  4.4031]               
## 
## Quantifying heterogeneity:
##  tau^2 = 1.1544 [0.3300; 16.7708]; tau = 1.0744 [0.5744; 4.0952]
##  I^2 = 95.2% [90.7%; 97.5%]; H = 4.58 [3.28; 6.38]
## 
## Test of heterogeneity:
##      Q d.f.  p-value
##  62.80    3 < 0.0001
## 
## Details on meta-analytical method:
## - Inverse variance method
## - Restricted maximum-likelihood estimator for tau^2
## - Q-Profile method for confidence interval of tau^2 and tau
## - Hedges' g (bias corrected standardised mean difference; using exact formulae)
```

```
#Random Effect
rm.raw <- metacont(Ne,
                  Me,
                  Se,
                  Nc,
                  Mc,
                  Sc,
                  data=rawdata,
                  studlab=paste(Study),
                  comb.fixed = FALSE,
                  comb.random = TRUE,
                  hakn = FALSE,
                  prediction=TRUE,
                  sm="SMD")
rm.raw
```

```
## Number of studies combined: k = 4
## Number of observations: o = 358
## 
##                          SMD            95%-CI     z p-value
## Random effects model -0.7926 [-1.8730; 0.2878] -1.44  0.1505
## Prediction interval          [-5.9883; 4.4031]              
## 
## Quantifying heterogeneity:
##  tau^2 = 1.1544 [0.3300; 16.7708]; tau = 1.0744 [0.5744; 4.0952]
##  I^2 = 95.2% [90.7%; 97.5%]; H = 4.58 [3.28; 6.38]
## 
## Test of heterogeneity:
##      Q d.f.  p-value
##  62.80    3 < 0.0001
## 
## Details on meta-analytical method:
## - Inverse variance method
## - Restricted maximum-likelihood estimator for tau^2
## - Q-Profile method for confidence interval of tau^2 and tau
## - Hedges' g (bias corrected standardised mean difference; using exact formulae)
```

```
forest(rm.raw, prediction = FALSE, lab.e = "OSA",
       rightlabs = c("g","95% CI","weight"),
       leftlabs = c("Study", "N","Mean","SD","N","Mean","SD"), text.random = "Overall effect")
```

```
#Random Effect
rm1.raw <- metacont(Ne,
                  Me,
                  Se,
                  Nc,
                  Mc,
                  Sc,
                  data=rawdata,
                  exclude = c(1),
                  studlab=paste(Study),
                  comb.fixed = FALSE,
                  comb.random = TRUE,
                  hakn = FALSE,
                  prediction=TRUE,
                  sm="SMD")
rm1.raw
```

```
## Number of studies combined: k = 3
## Number of observations: o = 358
## 
##                          SMD              95%-CI     z p-value
## Random effects model -1.2232 [ -2.1822; -0.2643] -2.50  0.0124
## Prediction interval          [-13.2202; 10.7737]              
## 
## Quantifying heterogeneity:
##  tau^2 = 0.6521 [0.1332; 27.5242]; tau = 0.8075 [0.3649; 5.2463]
##  I^2 = 92.0% [79.7%; 96.8%]; H = 3.53 [2.22; 5.62]
## 
## Test of heterogeneity:
##      Q d.f.  p-value
##  24.95    2 < 0.0001
## 
## Details on meta-analytical method:
## - Inverse variance method
## - Restricted maximum-likelihood estimator for tau^2
## - Q-Profile method for confidence interval of tau^2 and tau
## - Hedges' g (bias corrected standardised mean difference; using exact formulae)
```

```
forest(rm1.raw, prediction = FALSE, lab.e = "OSA",
       rightlabs = c("g","95% CI","weight"),
       leftlabs = c("Study", "N","Mean","SD","N","Mean","SD"), text.random = "Overall effect")
```
